# Cognitive Biases in Cannabis, Opioid, and Stimulant Disorders: A Systematic Review

**DOI:** 10.3389/fpsyt.2018.00376

**Published:** 2018-08-15

**Authors:** Melvyn W. B. Zhang, Jiangbo Ying, Tracey Wing, Guo Song, Daniel S. S. Fung, Helen E. Smith

**Affiliations:** ^1^National Addiction Management Service, Institute of Mental Health, Singapore, Singapore; ^2^Family Medicine and Primary Care, Lee Kong Chian School of Medicine, Nanyang Technological University Singapore, Singapore, Singapore; ^3^National Psychiatry Residency Program, National Healthcare Group, Singapore, Singapore; ^4^Department of Developmental Psychiatry, Institute of Mental Health, Singapore, Singapore

**Keywords:** attention bias, approach bias, cognitive bias, substance use, addiction

## Abstract

**Background:** Opiates, cannabis, and stimulants are highly abused and are prevalent disorders. Psychological interventions are crucial given that they help individuals maintain abstinence following a lapse or relapse into substance use. The dual-process theory has posited that while the repeated use of a substance leads to increased automatic processing and increased automatic tendencies to approach substance-specific cues, in addition to the inhibition of other normal cognitive processes. Prior reviews are limited, as they failed to include trials involving participants with these prevalent addictive disorders or have not reviewed the published literature extensively.

**Objectives:** The primary aim of this review is to synthesize the evidence for cognitive biases in opioid use, cannabis use, and stimulant use disorders. The secondary aim of the review is to determine if cognitive bias could be consistently detected using the different methods. Lastly, this review will narratively synthesize the evidence of possible associations between cognitive biases and other addiction-related outcomes.

**Methods:** A search was conducted from November 2017 to January 2018 on PubMed, MEDLINE, Embase, PsycINFO, Science Direct, Cochrane Central, and Scopus. Articles were included if participants had a primary diagnosis of opioid use, cannabis use, or stimulant use disorder. The selection process of the articles was in accordance with the Preferred Reporting Items for Systematic Reviews and Meta-Analysis guidelines. A qualitative synthesis was undertaken.

**Results:** A total of 38 studies were identified. The main finding is the evidence that cognitive biases are present in the 38 studies identified, except for a single study on opioid use and stimulant use disorders. Cognitive biases were reported despite a variety of different methods being utilized. Synthesis of secondary outcome was not feasible, due to the varied outcomes reported.

**Conclusions:** Cognitive biases have been consistently observed in opioid use, cannabis use, and stimulant use disorders, despite a range of assessment tools being utilized in the assessment for these biases.

## Introduction

Attention bias in substance use disorder refers to the preferential allocation of attention to substance-related stimuli (1), while approach biases refer to the automatic action tendencies to reach out for substance-related cues (1). Thus, this implies that for an individual with a substance use disorder, substance-related cues can grab the individual's attention (2). Previously theories such as classical conditioning or incentive-sensitisation theories, have provided explanations as to how these cognitive biases develop (2). More recently, the dual-process theory has posited that whilst the repeated usage of a substance leads to increased automatic processing and increased automatic tendencies to approach substance-specific cues, in addition, other normal cognitive processes are affected (3). The cognitive control process, which normally serves to inhibit this automatic behavior, is itself inhibited (3). This coupled with the increased dopamine in the cortico-striatal circuit, results in individuals having increased attention and automatic tendencies toward substances, precipitating a relapse. Cox et al. (4) highlighted the need for specific interventions to modify these automatic unconscious processes. Whilst the Stroop task has been routinely used for the assessment of cognitive biases, several other paradigms have been used to retrain attentional or approach tendencies, such as the visual probe or the approach/avoidance task (4, 5). The visual probe task, when used as a cognitive retraining intervention instead of an assessment intervention, involves pairing probes with a neutral stimulus 100% of the time, to retrain attentional biases away from substance cues (4). When the approach/avoidance task is used as a retraining tool, it involves the presentation of substance-related cues in the push-away format and neutral cues in a pull-closer format (5). To date, there has been some initial research conducted on substance use disorders, both in determining whether cognitive biases are present for specific addictive disorders and in the evaluation of the effectiveness of bias modification (6, 7).

Several reviews have examined the presence of cognitive biases in addictive disorders as well as the effectiveness of bias modification. Cristea et al. (1) undertook the first meta-analysis examining the effectiveness of bias modification for substance use disorders. Even though the authors included in their review search terminologies for a variety of addictive disorders, they only managed to identify and include studies that included participants with alcohol or tobacco use disorders. This review reported that bias modification for both cognitive biases was moderately effective, with an effect size of 0.60 (Hedge G) (1). Notably, the review found no association between the reduction in biases and other outcomes such as cravings. One of the major limitations of the review (1) was that it included participants who had either alcohol or tobacco disorders, with no studies being included that focused on other substances of abuse. High risk of biases was identified in the studies include, which might have affected the results synthesis. Whilst Cristea et al. (1) did not, in their meta-analytical review found any association between cravings and attentional biases, their findings are not unexpected. Field et al. (8) in their previous review sought to determine the relationship between cravings and attentional biases. From the 68 studies that they have included, they reported the presence of a weak relationship between attentional biases and cravings (*r* = 0.19). Field et al. (8) have reported there being a larger association between attentional biases and illicit substances and caffeine, as compared to alcohol and tobacco. Christiansen et al. (9) attempted in their review to determine if there was an association between cognitive biases and relapse, and their study included participants with alcohol, tobacco, cocaine, and cannabis disorders. It was reported in the review that the assessment of cognitive biases did not predict relapse and that attentional bias modification did not reduce the future risk of relapse. Whilst the review by Christiansen et al. (9) included participants with a range of addictive disorders, the time frame was not specified, nor the inclusion and the exclusion criteria used in the selection of the articles. In addition, the database search was limited to that of PubMed, Scopus, and prior published reviews. Hence, there is a possibility that other relevant citations were missed and reduced the quality of the evidence synthesis.

The fact that the reviews to date are limited to certain substance disorders matters. The recent report released by the United Nations Office on Drugs and Crime (UNDOC) stated that in 2015, 29.5 million individuals had a substance use disorder (10), with an estimated 28 million healthy years of life lost due to drug use (10). Substances like cannabis, opioids, amphetamines, and prescription opioids are widely abused. In 2015, it was estimated that there were 183 million cannabis users, 35 million opioid users, and 37 million amphetamine and prescription stimulant users (10). For the management of substance use disorders, there are limited pharmacological options. Symptomatic medications, such as benzodiazepines or antipsychotics, are routinely prescribed for patients to control acute intoxication symptoms following cannabis or stimulant use. There remain no medications that are approved to help individuals maintain abstinence following cannabis or stimulant use. Psychological interventions can help individuals maintain abstinence following a lapse or relapse (11). Psychological interventions routinely used include cognitive behavioral therapy, contingency management, and more recently mindfulness-based relapse prevention (11). A prior review provided evidence that cognitive behavioral therapy is effective for substance use disorders, with an effect size of 0.45 (Cohen D) (12). However, despite this effectiveness of cognitive behavioral therapy, 40–50% of individuals still relapse within a year of successful treatment and another 70% of individuals relapse within 3 years (13). The high relapse rate following a moderately successful intervention suggests that it has not adequately addressed all the issues leading to a lapse or relapse. Cognitive behavioral therapy mainly addresses the cognitive control issues and fails to address the unconscious processes responsible for relapse. Consequently, further studies have been conducted evaluating the effectiveness of cognitive bias modification for individuals with different types of substance use disorders.

Given the limitations of previous reviews and the recent review by (14), together with recent studies that have evaluated cognitive biases in substance disorders that are highly prevalent (opioid, cannabis, and stimulant use disorders), there is a need for a systematic review to synthesize the information from these studies. The primary aim of this review is to synthesize the evidence for cognitive biases in the following substance use disorders: opioid use, cannabis use, and stimulant use disorders. If biases are consistently present, this will help to guide future research involving bias modification. The secondary aim of the review is to determine if cognitive bias could be consistently detected using the different methods. Lastly, this review will narratively synthesize the evidence of possible associations between cognitive biases and other addiction-related outcomes.

## Methods

A search was conducted, on the following databases (PubMed, MEDLINE, Embase, PsycINFO, Science Direct, and Scopus), from inception through to January 2018. The following search terminologies were used: (“attention bias” OR “approach bias” OR “avoidance bias” OR “cognitive bias”) AND (“addiction” OR “substance” OR “drug” OR “abuse” OR “dependence” OR “Opiates” OR “Heroin” OR “Cannabis” OR “Marijuana” OR “Stimulants” OR “Amphetamines” OR “Cocaine”).

### Inclusion and exclusion criteria

Only articles that were written in English language were included. Articles were included if participants had a primary diagnosis of opioid use, cannabis use, or stimulant use disorder. Articles were excluded if participants had other psychiatric disorders as a primary disorder, or if the study involved a pharmacological intervention in which medications were used to examine their effects on cognitive biases. All types of study design were included.

### Selection of articles

Two authors (MZ and JY) independently selected the relevant articles. Articles were initially screened based on their titles and abstracts. Articles that were shortlisted were then further evaluated against the inclusion and exclusion criteria. If the reviewers disagreed, this was resolved through a discussion with another author (GS.). The selection of the articles for inclusion was in accordance with the Preferred Reporting Items for Systematic Reviews and Meta-Analysis Guidelines.

### Statistical analysis

The following data were systematically extracted from each of the identified articles and recorded on a standardized electronic data collation form: (a) authors and study year, (b) study design and methodology (study design, sample size, types of sample, country, demographics of sample, diagnosis of participants, methods in which diagnosis was established), (c) cognitive biases assessment and modification methods, (d) outcomes of interest (presence of attention or approach biases, effectiveness of biases modification, other secondary outcomes reported such as craving scores or addiction outcomes).

As the reported outcome measures were heterogeneous, a meta-analytical synthesis was not feasible. The secondary outcomes reported varied by the studies, and even if there were similar outcomes reported, very often, a quantitative approach (meta-analysis) was not feasible as no effect size or statistics were reported. Hence, a qualitative synthesis was undertaken instead. As both randomized and non-randomized studies were included, there remains no single risk of bias tool that could be applied across all the studies for quality assessment. Hence, quality assessment was not performed.

## Results

**Figure 1:**
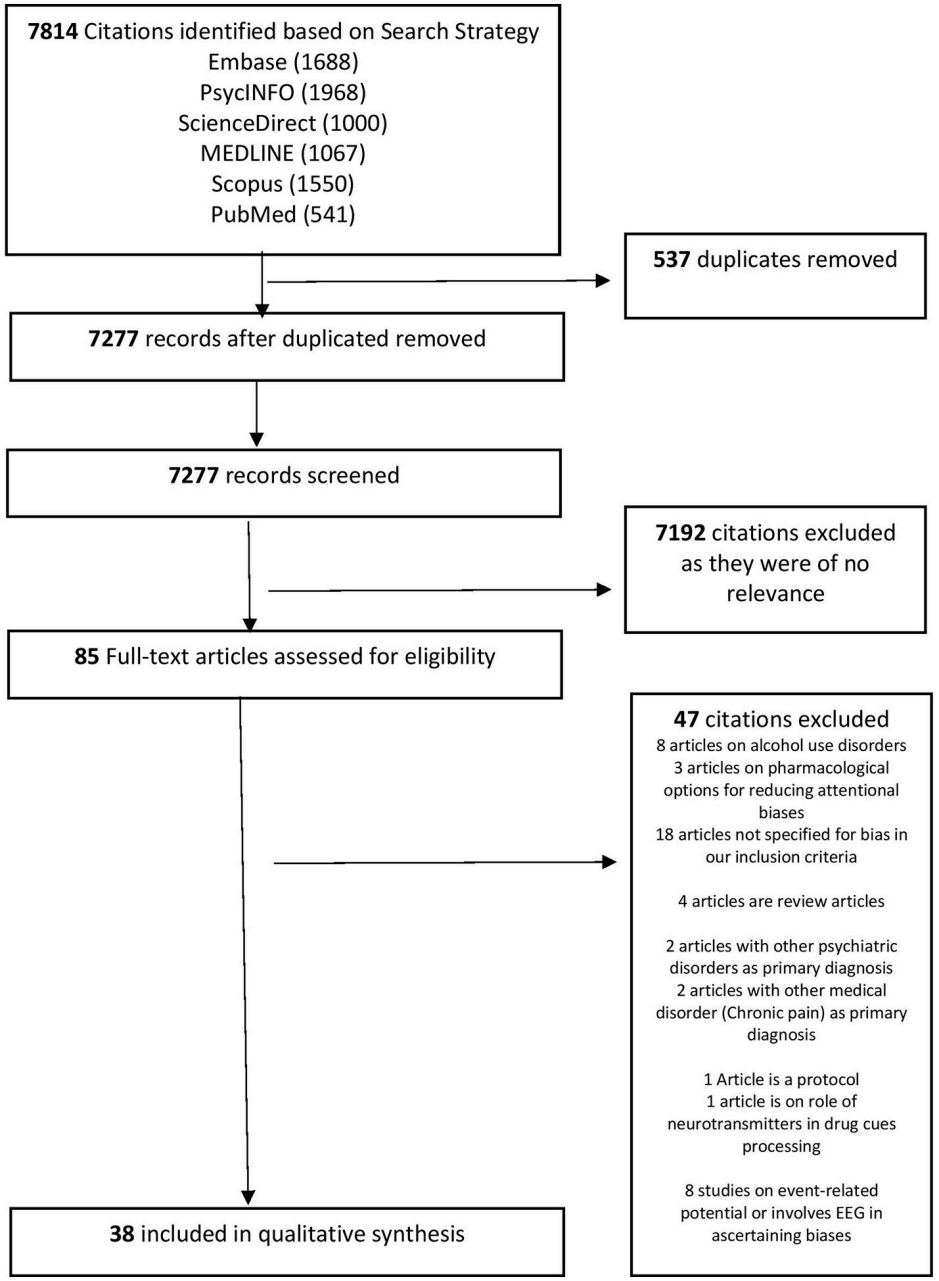
Flowchart for the Selection of Articles.

Based on our search strategy, a cumulative total of 7,814 citations were extracted from Embase, PsycINFO, Science Direct, Scopus, MEDLINE, and PubMed. Five hundred and thirty-seven duplicated articles were removed, leaving 7,277 citations. On further screening, 7,192 citations were removed as they were of not relevant. Eighty-five full-text articles were downloaded and evaluated against the inclusion and exclusion criteria. Forty-seven citations were excluded for reasons documented in Figure 1. Thirty-eight articles were selected for this qualitative synthesis. Figure 1 provides an overview of the selection process of the articles for the current review. Tables 1–4 provide an overview of the core characteristics of the included studies (*n* = 38).

**Table 1:** Characteristics of studies for opioid use disorders.

**Study**	**Study design**	**Sample size**	**Types of sample**	**Demographics of sample**	**Country**	**Diagnosis of sample**	**Method of diagnosis**	**Attention bias method**	**Outcomes**
**OPIOID USE DISORDERS**									
Franken et al. (15)	Randomised trial	21 heroin dependent participants 30 control participants	Participants with heroin dependence were recruited from an inpatient treatment center Participants in the control group were recruited among clinical and administrative staff in the clinic	71.4% males (heroin dependent) 83.3% (control group) Mean age 31.5 years (heroin dependent) Mean age 34.8 (control group) Mean self-reported duration of heroin dependence was 93.9 months	Netherlands	Heroin Dependence	Based on the DSM-IV criteria for heroin dependence	Drug Stroop task	Higher overall reaction time for heroin participants as compared to control participants Mean pre-experimental craving was 13.8, mean post masked Stroop was 7.19 and mean post unmasked Stroop was 15.2
Lubman et al. (16)	Randomised trial	16 methadone maintained opiate addicts 16 age-matched control	Heroin addicts were recruited from local drug services Staff from these services were recruited as controls	Opiate group: mean age 31.4, Male to Female 11:5 Control group: mean age 31.7, male to female 8:8	United Kingdom	Heroin Dependence	ICD-10 and DSM IV	Pictorial Probe Detection Task	Faster reaction times to probes that replaced drug stimuli, indicative of the presence of an attentional bias
Marissen et al. (17)	Randomised trial	110 Participants assigned to either cue exposure therapy or placebo psychotherapy	Abstinent heroin addicts who were admitted voluntarily to an in-patient drug-free therapeutic center in the Hague	89% males, mean age 34 years old Average age of onset of heroin usage was 21.4 years, most have used heroin for 9.3 years	Netherlands	Heroin Dependence	DSM-IV criteria for heroin dependence	Emotional Stroop Task	Pre-treatment attentional bias predicted relapse at 3 months follow-up Reduction of attentional biases in both experimental conditions
Bearre et al. (18)	Cross-sectional study	60 participants	Recruited from a harm reduction program (methadone maintenance or needle exchange)	44 participants on methadone maintenance, 16 on needle exchange program Mean age 32 years, mean 7.07 months of heroin use	United Kingdom	Heroin dependence	Not mentioned	Flicker change blindness paradigm	Attention bias increase as the monthly frequency of heroin use increases
Fadardi et al. (19)	Cross-sectional study	53 drug abusers with 71 non-abusers as controls	Drug abusers were recruited from a local drug-use services clinic and were on methadone maintenance therapy Controls were students, academic staff and other personnel of Ferdowsi University of Mashhad and people from the local community who were visiting the university	Drug abusers: 100% male, mean age was 36.57 years Control: 54% male, mean age 26.62 years	Iran	Opiate abuse	Not mentioned	Persian Version of classic and addiction Stroop tests	Higher attentional bias for drug-related stimuli than non-abusers
Waters et al. (20)	Longitudinal study	68 heroin dependent inpatients	Addiction treatment center	85.3% Males Mean age 40.87 Age of first heroin use was 22.34 Total years of heroin use was 14.13	Netherlands	Heroin Dependence	DSM-IV criteria for heroin dependence	Drug Stroop task	Attentional biases to drug cues were elevated during temptation episodes
Zhou et al. (21)	Cross-sectional study	22 male abstinent heroin abusers 20 healthy males	Recruited from a legal rehabilitation center in Yunnan Province, China	All males Mean age 31.45	China	Opioid dependence	DSM-IV criteria for heroin dependence	Pull/Push task (Based on Approach/Avoidance Task rationale)	Abstinent individuals had higher tendencies to approach heroin-related stimuli, and avoidance tendencies toward heroin stimuli were reduced
Anderson et al. (22)	Cross-sectional study	17 opioid dependent patients 17 controls	Opioid dependent patients were from local methadone clinic Controls recruited from newspaper advertisements and flyer postings	Patients: mean age 44.4; male to female 9:8 Control: mean age 42.9; male to female 9:8	United States	Opioid Dependence	Structured clinical interview for DSM-IV (SCID-I)	Attentional Visual Search Task	Patients in treatment for addiction experience greater difficulty ignoring stimuli associated with non-drug reward.
Charles et al. (23)	Cross-sectional study	23 opioid dependent 21 healthy controls	Those prescribed with opioid substitution within the National Health Service	Mean age for Patient group (ABM-away) 43.91, (ABM-control) 45.17 Control group (ABM-away) 41.00, ABM-Control 38 Gender M:F for patient group ABM-away 10:1, ABM-control 10:2 Control group ABM-away 8:3, ABM-contor 7:3	United Kingdom	Opioid Dependence	Not mentioned	Visual Probe Tasks	No baseline differences in attentional biases between control and patient group No change in AB following bias modification Treatment adherent patients who did not use illicit opiates on top of their prescribed opiates had statistically significantly greater attention bias away from substance-related stimuli
Ziaee et al. (24)	Cross-sectional	24 in experimental group received Drug Attention Control Training Program in addition to treatment as usual 24 in control group received only treatment as usual	Participants were drug abusers who were undergoing methadone maintenance therapy in a drug-abuse clinic	Mean age of experimental group was 33.17; mean age of control group was 38.75 The drugs that participants most commonly abused were opium, crystal and opium sap	Iran	Drug abuse and on methadone maintenance therapy	Not mentioned	Drug Stroop test	Experimental group showed reduction in attention bias for drug related stimuli, temptations to sue, doses of medicine, and number of relapses; increases on the Readiness to Change Questionnaire and 2 subscales of the Situational Confidence Questionnaire
Zhao et al. (25)	Cross-sectional	30 heroin dependents 39 healthy controls	Heroin dependents were from a methadone clinic Controls were service workers and security guards from a university	In heroin group: mean age 39.83, male to female 2:1 In control group: mean age 41.42, male to female 9:10	China	Heroin dependence	DSM-IV	Visual probe task with concurrent eye movement monitoring	Heroin group reacted faster to probes associated with substance-related pictures than neutral pictures, and they directed more initial fixations and maintained longer initial fixation durations toward substance-related pictures than neutral pictures.

**Table 2:** Characteristics of studies for cannabis use disorders.

**Study**	**Study design**	**Sample size**	**Types of sample**	**Demographics of sample**	**Country**	**Diagnosis of sample**	**Method of diagnosis**	**Attention bias method**	**Outcomes**
**CANNABIS USE DISORDERS**									
Field et al. (26)	Case control	17 regular cannabis users 16 non-users	Students and Staff at the University of Southampton	5 males, 12 females in the group of cannabis users Mean age of 22.4 years Median length of time cannabis smoked 3 years 4 males, 12 females in control group. Mean age 20.9 years.	United Kingdom	Cannabis	No mention of any diagnostic tools	Visual Probe Task	High levels of craving associated with significant attention bias for cannabis-related words
Field et al. (27)	Cross-sectional study	28 recreational cannabis users, including 15 in the “dependent” group and 13 in the “non-dependent” group	Students or staff at the University of Liverpool	6 female, 22 male Mean age was 21.9 years	United Kingdom	Cannabis Dependence	Scores on the Cannabis Severity Dependence Scale	Drug Stroop task	Significant attention bias in the cannabis group No association between attention bias and frequency of cannabis use or subjective craving
Field et al. (28)	Case control	23 regular cannabis users 23 non-user controls	Students and staff at the University of Liverpool	Cannabis users mean age was 23.04 Non-users mean age was 21.30 Gender ratio for Cannabis users: 14:9 (M:F) Gender ratio for non-users 8:15 (M:F)	United Kingdom	Cannabis abuse	Self-report usage of drugs	Visual Probe task with concurrent eye movement monitoring	Regular cannabis users had biases to maintain gaze on cannabis cues, and faster approach responses to cannabis cues
Cane et al. (29)	Cross-sectional study	17 marijuana-smokers 15 non-marijuana smokers	Students from the University of Kent	Not provided	United Kingdom	Cannabis dependence	Screening questionnaire	Drug Stroop task	Mean reaction time was significant for the marijuana group (Mean time for marijuana words was 984ms, neutral words was 895ms).
Cousijin et al. (30)	Cross-sectional study	32 heavy cannabis users and 39 non-using controls	Recruited through advertisements on the Internet and in Cannabis outlet (coffee-shops)	34% females for heavy cannabis users, mean age 21.2 37% female for controls, mean age 22.0	Netherlands	Cannabis dependence	Cannabis Use Disorder Identification Test and Structured Diagnostic Interview (MINI)	Approach and Avoidance Task	Heavy cannabis users had approach bias for cannabis as compared to controls. Approach bias was predictive of cannabis use at 6 months follow-up.
Cousijn et al. (31)	Cross-sectional Study	42 heavy cannabis users with intentions to use 45 heavy cannabis users shortly after cannabis usage	Recruited in five different cannabis outlets	82 males, 8 females Aged 18-59	Netherlands	Cannabis dependence	Cannabis Use Disorder Identification Test-Revised	Approach and Avoidance Task	Heavy cannabis users with the intention to use did not show a cannabis approach bias, whereas intoxicated cannabis users did show an approach bias regardless of image category. Moreover, craving was negatively associated with the approach bias, and no relationships were observed between the cannabis approach bias, satiation, prior cannabis use, and response inhibition
Cousijin et al (32)	Cross-sectional study	27 heavy cannabis users 26 controls	All recruited via Internet advertisement and Amsterdam coffee shops	30% females in cannabis group, 38% females in control group Mean age 24.0 years in cannabis group Mean age 25.3 years in control group	Netherlands	Cannabis dependence	Cannabis Use Disorder Identification Test-Revised	Classical and Cannabis Stroop test	Cannabis smokers as compared to control group have stronger attentional bias for cannabis word Within the group of cannabis users, those who were clinically recognized as dependent showed a stronger attentional bias than the heavy, non-dependent users. Cannabis users who displayed reduced cognitive control (as measured with the classical Stroop task) showed increased session-induced craving. Cognitive control did not appear to modulate the relationship between attentional bias to cannabis words (cannabis Stroop task) and cannabis dependence.
Metrik et al. (33)	Cross-sectional	93 participants	Recruited from community	34.4% female	United States	Cannabis dependence	SCID-IV-NP for assessment of cannabis dependence	Marijuana Stroop Test	Presence of Attention bias to marijuana words (mean time was 752.89) vs. control (721.88) *p* < 0.001
Vujanovic et al. (34)	Cross-sectional	12 adults with cannabis use disorders 13 controls	Recruited via local advertisements	8 female Mean age 31, range 22-45	United States	Cannabis use disorder	Screening test and structured clinical interview for DSM-IV Axis I disorder	Pictorial stimuli attention bias task	Cannabis use disorders group showed greater attentional bias to cannabis cues at the 125-ms probe time Cannabis use disorders group also reported greater perceived stress and post-task stress scores

**Table 3:** Characteristics of studies for stimulant use disorders.

**Study**	**Study design**	**Sample size**	**Types of sample**	**Demographics of sample**	**Country**	**Diagnosis of sample**	**Method of diagnosis**	**Attention bias method**	**Outcomes**
**STIMULANT USE DISORDERS**									
Franken et al. (35)	Pilot Study	16 cocaine dependence or cocaine abuse individuals	Addiction treatment department of a psychiatric hospital	13 males, 3 females Mean age was 26.4 years Mean cocaine abstinence was 184 days Mean age of first cocaine use was 18.6 years Mean years of regular cocaine use was 5.7 years	Netherlands	Cocaine abuse/dependence	DSM-III-R criteria for cocaine dependence or cocaine abuse	Reaction time experimental task	Attentional bias was present in patients with higher scores on the obsessive cocaine thoughts and higher craving scores.
Hester et al. (36)	Case control	23 cocaine users with 23 matched controls	Not available	23 non-drug using participants (7 female), mean age 39.4 years 23 active drug using participant (7 female, mean age 40.3)	United States of America	Cocaine dependence	Not available	Emotional Stroop Task	Mean reaction time for cocaine words for cocaine users (922.7ms), for controls (772.2ms). Significant attention bias for active cocaine users
Vadhan et al. (37)	Cross-sectional study	17 cocaine-dependent treatment seeking males 20 cocaine-dependent nontreatment seeking males	Recruited from two clinical treatment trials of combination pharmacotherapy and cognitive-behavioral relapse prevention therapy Non-treatment seekers were recruited from behavioral pharmacology studies	Mean age of treatment seekers was 42.2, mean age of nontreatment seekers was 40.4	United States	Cocaine Dependence	DSM-IV criteria, Structured Clinical Interview	Drug Stroop task	Treatment seeking participants have increased response latency when presented with cocaine-related words. Non-treatment seeking participants did not demonstrate this.
Hester et al. (38)	Cross-sectional Study	16 active cocaine users	No mention of source of recruitment, but participants were retained in the study if they had used cocaine in the past 72 h.	Mean age 41, Mean education 11.6 years	Australia	Cocaine dependence	Not mentioned	Working Memory task that manipulated attention by varying behavioral contents	Cocaine users had significantly poorer attentional control under high working memory demands, suffering both increased response times and reduced recall accuracy, with this effect more pronounced for cocaine stimuli
Montgomery et al. (39)	Randomised trial	32 regular cocaine users and 40 non-users	Student Population at Liverpool John Moores University and the general population in the surrounding areas	Mean age for cocaine users assigned to placebo 19.29, assigned to alcohol 20.23 Mean age for non-users assigned to placebo 19.59, assigned to alcohol 20.0 13 male in cocaine use group 19 male in non-cocaine use group	United Kingdom	Cocaine dependence	Questionnaire	Visual Probe and Modified Stroop task	Cocaine participants who received alcohol had increased attentional bias for cocaine pictures The cocaine Stroop revealed no differences between cocaine users and non-users, and no effects of alcohol in either group.
Liu et al. (40)	Cross-sectional study	37 cocaine-dependent subjects, 32 controls	Recruited from an ongoing neuroimaging study and via newspapers advertisements	Cocaine dependent subjects used cocaine for a mean of 13.64 years	United States	Cocaine dependence	Structured clinical interview for DSM-IV (SCID-I)	Cocaine Stroop Task	Cocaine-dependent subjects showed attentional bias to cocaine-related words, increased impulsivity, and poor inhibitory control compared with controls. The attentional bias was associated with inhibitory control in cocaine-dependent subjects but not in control subjects.
Tull et al. (41)	Cross-sectional study	30 cocaine dependent patients with PTSD, 30 cocaine dependent without PTSD	Residential substance use disorder treatment center	Mean age cocaine with PTSD was 44.57,cocaine without PTSD was 44.27 Male in cocaine with PTSD was 26.7%, in cocaine without PTSD 83.3%	United States	Cocaine Dependence	Not mentioned	Visual Dot probe task	Differences in attentional bias following script intervention (neutral or trauma script) Non-PTSD participants have greater attentional bias following neutral script than PTSD participants This effect was reversed following trauma script exposure, with PTSD participants exhibiting a greater attentional bias toward the location of cocaine imagery than non-PTSD participants.
Carpenter et al. (42)	Cross-sectional Study	25 individuals	Active cocaine users seeking treatment at Columbia University's Substance Treatment and Research Service	Mean age 37 years	United States	Cocaine dependence	Structured clinical interview for DSM-IV (SCID-I) and independent psychiatric examination	Drug Stroop	Attention bias toward cocaine related stimuli not demonstrated Stronger implicit beliefs about the positive effects of cocaine use prior to treatment were associated with poorer treatment outcome when an escalating voucher-incentive program was in place. An attentional bias for cocaine-related stimuli was associated with better treatment outcome when an escalating voucher-incentive program was removed. No association between cocaine use beliefs and treatment outcome was found when beliefs were measured with self-report instruments.
Marhe et al. (43)	Cross-sectional study	34 cocaine dependent inpatients	Recruited from an addiction treatment center	Mean age 38.7 85% males 11.4 years of using cocaine	Netherlands	Cocaine dependence	DSM-IV criteria for cocaine dependence	Cocaine Stroop test	Presence of attentional bias, with cocaine dependent patients responding slower to cocaine stimuli
Bardeen et al. (44)	Cross-sectional study	22 cocaine dependent patients with BPD 36 cocaine dependent patients without BPD	Community based residential substance use disorder treatment facility	26 females, mean age of 44.5 years	United States	Cocaine dependence	DSM-IV criteria for cocaine dependence	Dot-probe task	Males with BPD have higher attentional bias scores following a post trauma script intervention
Kennedy et al. (45)	Cross-sectional study	35 individuals	Substance abuse and treatment program	20 non relapsers, mean age 43.7 15 relapsers, mean age 43.0	United States	Cocaine dependence	Structured clinical interview for DSM-IV	Stroop tasks	Attention bias was present, with cocaine-dependent subjects having increased reaction time for personal drug use words (928.6ms) vs. neutral words (897.2) The level of attentional bias for cocaine-use words was not predictive of eventual relapse
Dias et al. (46)	Cross-sectional study	46 cocaine dependent subjects 41 healthy controls	Recruited by a variety of media advertisements	cocaine dependent group Mean age was 46.3 Control mean age was 40.0 cocaine dependent group %Male was 84.8%, Control %Male was 51.2	United States	Cocaine dependence	DSM-IV (SCID-1)	Eye-tracking cocaine attentional bias task	Presence of attentional bias toward cocaine cues as cocaine dependent subjects made more anti-saccade to cocaine cues
Mayer et al. (6)	Randomised trial	37 participants Assigned to either attentional bias modification therapy (ABMT) or control therapy	Not mentioned	ABMT group: 14male, and 5 female, mean age 37.4 Control group: 10 male and 8 female, mean age 38.9	United States	Cocaine dependence	Structured clinical interview for DSM-IV	Visual Probe task	Presence of attentional bias Not subjected to modification byABMT
Marks et al. (47)	Cross-sectional study	20 cocaine dependence 20 cocaine and alcohol dependent	Recruited through word of mouth and postings on community bulletin boards	Cocaine mean age 43.4 Cocaine alcohol mean age 43.4 6 females in each groups	United States	Cocaine and alcohol dependent	Structured clinical interview for DSM-IV	Eye-tracking cocaine attentional bias task	Cocaine dependent had attentional bias toward cocaine Cocaine-alcohol dependent has attentional bias toward both cocaine and alcohol.
Devito et al. (48)	Randomised trial	38 in treatment as usual plus computer-based CBT (CBT4CBT) 41 in treatment as usual	Recruited from community based outpatient clinic	46% female, mean age 42.2	United States	Cocaine use disorder	DSM-IV	Computerized drug Stroop test	Reductions in Drug Stroop Effect across treatment were associated with greater engagement with CBT4CBT-specific treatment components
Sharma et al. (49)	Cross-sectional study	Study 1 52 participants, 27 of which were regular marijuana users and 25 were not marijuana users Study 2 16 participants	Recruited through social networks and known to be marijuana smokers or non-users Recruited from two local Narcotics Anonymous Fellowships	Mean age 31 years old 38 males 13 males, 3 females, with ages of 25 to 41	United Kingdom	Cannabis dependence Cocaine dependence	Self-report Self-report	Drug Stroop task	A slowdown in responding to the color of non-words that were paired with cocaine-related images compared with non-cocaine related images. The slowdown was also characterized as a carryover effect, with the largest effect occurring on trials following the addiction-associated non-word. No effects were found for marijuana images associated with non-words.

**Table 4:** Characteristics of studies with heterogenous sample of participants.

**Study**	**Study design**	**Sample size**	**Types of sample**	**Demographics of sample**	**Country**	**Diagnosis of sample**	**Method of diagnosis**	**Attention bias method**	**Outcomes**
**MIXED COHORT**									
Carpenter et al. (50)	Cross-sectional study	80 drug dependent individuals	All were illicit drug users enrolled in one of the six randomized trial combining coping skills relapse prevention and pharmacotherapy	62 males, 18 females 45 participants (cocaine), 25 participants (marijuana), 10 participants (heroin) Mean age 39.9 years	United States of America	Cocaine, Marijuana and Heroin dependence	DSM-IV dependence criteria Structured psychiatric interview	Drug Stroop Test	All groups had slower reaction times when presented with cocaine words.
Van Hamel et al. (51)	Cross-sectional	78 adolescents with substance dependence 64 healthy controls	Addiction care facility	Substance dependence group mean age 19.5 Control group mean age 19.0	Netherlands	Alcohol, Cannabis, Amphetamine or GHB Use disorder	Self-reported substance usage and severity of usage	Visual Probe task	Attentional bias for stimuli presented at 500ms and 1250ms. Higher severity of dependence, increased AB

Eleven articles involved participants with opioid use disorder, 16 articles involved participants with cocaine use disorder, nine articles involved participants with cannabis use disorder, and two articles involved samples of participants with different disorders (i.e., drug-dependent participants with either cocaine, cannabis, or opiate dependence; or participants with alcohol, cannabis, amphetamine, or GHB use disorders). Out of the 11 articles involving participants with opioid use disorder, three were randomized trials, seven were cross-sectional studies and one was a longitudinal study. Out of the 16 articles involving participants with cocaine use disorder, three were randomized trials, 11 were cross-sectional studies, one a pilot study and one a case-controlled study. Out of the nine articles involving participants with cannabis use disorder, two were case-control studies and seven were cross-sectional studies.

### Characteristics of 11 studies for opioid use disorders

Three studies [Franken et al. (15), Marissen et al. (17), and Zhou et al. (21)], recruited participants in an inpatient treatment facility. The remaining studies (*n* = 8) recruited participants who were outpatients or who had attended a harm reduction program. Six studies [Bearre et al. (18), Fadardi et al. (19), Anderson et al. (22), Charles et al. (23), Ziaee (24), and Zhao et al. (25)] included participants who were receiving methadone substitution-based treatment. Apart from the study by Ziaee et al. (24), which failed to provide information about the gender ratios of the recruited cohort of individuals, all studies had predominantly male participants, and the mean age was between 30 and 45 years. Most of the included studies were conducted in Europe (*n* = 6), with the remaining studies conducted in the United States (*n* = 1), Iran (*n* = 2), and China (*n* = 2). The diagnosis of opioid use disorders was ascertained by means of the Diagnostic and Statistical Manual (DSM) criteria for almost all of the studies. Only four studies [those of Bearre et al. (18), Fadardi et al. (19), Charles et al.(23), and Ziaee et al. (24)] failed to report how the diagnosis was ascertained. In the assessment of attention biases, the Stroop task was used in five studies; the visual probe task in two studies and the remaining studies used the visual probe task with concurrent eye tracking (*n* = 1), approach and avoidance task (*n* = 1), attentional visual search (*n* = 1), and flicker change blindness paradigm (*n* = 1).

### Primary and secondary outcomes reported in the 11 studies for opioid use disorders

Ten of the eleven reported the presence of biases in their sampled participants, except in Charles et al. (23) reported no baseline attentional differences between the healthy controls and opioid-dependent participants. Studies that assessed attention bias using the Stroop task reported an higher overall reaction time (15) among abusers, or enhanced attentional biases for drug-related stimuli especially among abusers (19) and that attentional biases to drug-related cues were heightened when abusers had temptation episodes (20). Studies that assessed attention bias using the visual probe task reported that the reaction time for abusers was faster when they had probes that replaced drug-related stimuli. The only study that paired the visual probe task with concurrent eye tracking (25) demonstrated that abusers not only have faster reaction time to drug-related probes, but also more initial fixations and maintained fixations on drug-related probes or images. For the approach and avoidance task, Zhou et al. (21) reported that former users of opioids have a greater tendency to approach heroin-related stimuli and a reduced tendency to avoid or push away these stimuli. The study using the flicker change blindness paradigm reported an association between the attention biases and the monthly frequency of heroin use. The study assessing attentional bias using attentional visual search showed that attention biases were present and that the presence of attentional biases would cause abusers to have heightened attention for other non-drug-related reward probes.

Four of the included studies reported other secondary outcomes following attention bias assessment and modification and provided evidence for the potential effectiveness of bias modification. Franken et al. (15) reported a reduction in the mean craving scores following masked Stroop intervention. The study by Ziaee et al. (24) found bias modification was associated with a reduction in several outcome parameters, including temptations to use, doses of medication, and number of relapses. Marissen et al. (17) reported findings similar to those of Ziaee et al. (24) concerning relapse, as Marissen et al. (17) found that baseline attentional biases could predict relapse at three months' follow-up. With regard to the effectiveness of the interventions, Marissen et al. (17) reported a reduction in attentional biases amongst individuals who received either cue exposure therapy or placebo psychotherapy. In contrast to the reductions in attentional biases were demonstrated by Marissen et al. (17) and Charles et al. (22) did not find any changes in attentional biases following their intervention.

### Characteristics of 9 studies for cannabis use disorders

Four studies [Field et al. (26–28) and Cane e al. (29)] recruited students; the remainder (*n* = 5) recruited through advertisements or from cannabis outlets. No study recruited participants who were part of inpatient treatment or rehabilitation facility. The majority of the sampled participants were males. The mean age in years across all of the studies ranged from 20 to the early 30s. Seven of the studies were conducted in Europe, and the remaining two studies were conducted in the United States. There was heterogeneity in the method of ascertaining the diagnosis, with one study Field et al. (28) basing their diagnosis on self-reported information, four studies [Field et al., (27), Cane et al., (29), and Cousijin et al. (31, 32)] on questionnaires, and the remainder (Cousijin et al. (30), Metrik et al. (33), and Vujanovic et al. (34)] on structured interviews. In the assessment of biases, the Stroop task was used in four of the studies, and the remainder of the studies used the visual probe task (*n* = 1), or the visual probe task with concurrent eye tracking (*n* = 1), or the pictorial attention bias task (*n* = 1), or the approach and avoidance task (*n* = 2).

### Primary and secondary outcomes reported in 9 studies for cannabis use disorders

All of the studies provided evidence for the presence of biases. For study that assessed attention bias using the Stroop task, it was reported that the mean reaction times for cannabis or marijuana stimuli were longer as compared to neutral stimuli (29). Stroop-based testing also helped in the differentiation of users who used varying amounts of cannabis (32), as it was reported that those who were clinically dependent had had a stronger attentional bias as compared to those who were non-dependent. The visual probe task and the visual probe task coupled with concurrent eye tracking provided both direct and indirect evidence of the presence of attention bias, as regular cannabis users exhibited biases in maintaining gaze on cannabis cues and increased reaction times for cannabis cues (28). Like that of the visual probe task, the pictorial stimuli attention bias task also reported the presence of attentional biases among cannabis users and these biases were most prominent when the probe was presented for 125 ms (which is shorter than the normal duration of 500 ms). For the studies that used the approach and avoidance task, one study reported the presence of an approach bias amongst heavy users (individuals who used cannabis on 10 or more days per month), whereas the other study did not report the presence of an approach bias in heavy users but found an approach bias for cannabis cues only amongst those users who were intoxicated at the time of testing.

There is mixed evidence for the association between attention bias and cravings. Field et al. (26) reported that the high levels of cravings were associated with attentional biases but in a later study, Field et al. (27) reported observing no association between attention biases and cravings. Cousijin et al. (31) also reported the lack of an association between approach biases and cravings. One study Cousijin et al. (30) reported approach bias to be a predictor of relapse.

### Characteristics of the 16 studies for stimulant use disorders

Eight studies [Franken et al. (35); Vadhan et al. (37), Tull et al. (41), Carpenter et al. (42), Marhe et al. (43), Bardeen et al. (44), Kennedy et al. (45), and Devito et al. (48)] recruited a clinical or treatment-seeking cohort of participants, one study Montgomery et al. (39) recruited a student sample, and five studies [Hester and Garavan (38); Liu et al. (40), Dias et al. (46), Marks et al. (47), and Sharma et al. (49)] recruited a cohort of participants from the community. There was a predominance of males in those samples that reported a gender ratio. The mean age across the studies varied within the range of 20–45 years, with a single study Montgomery et al. (39) including an adolescent cohort (mean age 19 years old). Twelve of the included studies were conducted in the United States, four studies in Europe, and one study in Australia. The diagnosis of stimulant use disorder, most studies (12 out of 17 studies) used the diagnostic criteria, two studies used a questionnaire or self-report, respectively, and the remainder of the studies did not provide any information as to how the diagnosis was ascertained. Eight studies utilized Stroop testing, one study used a reaction time experimental task, one study used a working memory task, four studies used the visual probe task, and two studies used the visual probe task with eye-tracking.

### Primary and secondary outcomes reported in studies for stimulant use disorders

All of the included studies reported the presence of biases except Carpenter et al. (42), which failed to demonstrate an attention bias in their sample of cocaine users. Two studies reported the severity of the underlying dependency to affect the resultant biases [Franken et al. (35) and Van Hamel et al. (51)]. Studies that assessed attention bias using the visual probe with eye-tracking to assess for attentional bias demonstrated that the cocaine-using cohort exhibited gaze preferences toward cocaine stimuli.

Concerning secondary outcomes, Franken et al. (35) reported that attentional bias was elevated in patients who had higher levels of cravings. Liu et al. (40) observed that attentional bias amongst cocaine users was associated with heightened impulsivity and poorer inhibitory control. Marks et al. (47) reported that there appears to be generalization of attentional bias for cocaine stimuli to that of alcohol stimuli. Mayer et al. (6) reported that there was no effectiveness of attention bias modification in reducing cocaine-related attentional biases.

### Outcomes reported in studies with a heterogeneous sample

Two studies [Carpenter et al. (42) and Van Hamel et al. (51)] studied group with differing diagnosis. The studies included drug-dependent participants with either cocaine, cannabis or opiate dependence; or participants with alcohol, cannabis, amphetamines, or GHB use disorders. Both the Stroop test and the visual probe task were used in the assessment of attentional biases, and both trials demonstrated the presence of attentional biases.

## Discussion

This is perhaps the first review that synthesizes the evidence of cognitive biases in opiate, cannabis and stimulant use disorders. The main finding is the evidence that cognitive biases are present in the 38 studies identified, except for a single study on opioid use and stimulant use disorders. There were differences in the participants recruited for each disorder, with opioid use disorder studies recruiting mainly treatment-seeking individuals, who were either abstinent or maintained on opiate substitution therapy. The bias assessment methods used included the Stroop task, visual probe, eye movement tracking, approach and avoidance task, attentional visual search, and flicker change blindness paradigm. The findings for the secondary outcomes were narratively reported, but not synthesized due to their high heterogeneity.

Our main finding is the evidence that cognitive biases are present in opiate use, cannabis use, and stimulant use disorders. This finding is novel as there have not been any prior reviews of cannabis use disorders. In their meta-analysis, Cristea et al. (1) attempted to search for randomized trials for these substance disorders, but eventually only included trials that evaluated participants with alcohol or tobacco use disorders. Given this, the paper by Eberl et al. (5) only provided evidence that cognitive biases were present and could be subjected to modification among participants with either alcohol or tobacco use disorders. A review by Christiansen et al. (9) included participants with opiate use disorders (17, 42, 43) and participants with cocaine use disorders (17, 42, 43, 45, 52). Whilst the work by Field and Cox (2) provided evidence for the presence of cognitive biases in opiate and cocaine use disorders, the evidence synthesis was from a limited number of studies, given that the primary objective of the review was to ascertain the association between cognitive bias and relapse. In their critical review, Leeman et al. (53) provided evidence for the presence of attentional biases in cocaine use disorders. This review was limited only to the assessment of cocaine studies as the authors reported that prior studies have reported that individuals with cocaine use disorders have particularly robust cognitive biases and that, given the lack of effective treatment approaches, novel approaches such as bias modification need to be considered (53). It is clear that the current review addressed several gaps in the prior reviews, by synthesizing the evidence for cognitive biases in three highly prevalent substance use disorders (opioid, cannabis, and stimulant use disorders) through evaluating studies with different study designs. None of the prior reviews synthesized evidence for the presence of attentional biases for individuals with cannabis use disorders. Evidence synthesis for this is of particular importance, given that cannabis use has been increasingly globally (34), and an estimated 10% of regular cannabis users do eventually develop dependence. Also, apart from the risk of developing an addictive disorder, cannabis use has been associated with heightened risk for the development of other psychiatric disorders, such as psychosis, cognitive impairment, and potentially also amotivational syndrome. Thus, the identification of the presence of cognitive bias in individuals with cannabis use disorders signifies that there remains a need for interventions to deal with these unconscious processes. Also, cognitive bias assessment could also help in the differentiation of individuals who are clinically dependent on cannabis and those who are using cannabis recreationally, as it has been demonstrated that individuals who are clinically dependent have stronger attentional biases. Whilst our synthesized results demonstrate the presence of cognitive biases in all three addictive disorders, it should be noted that there were two outlier studies (23, 42). One of the studies (23) attributed this finding to the fact that the presentation of the opioid-related stimuli might have appeared to be novel to the control sample.

Our synthesis of the published literature demonstrated the presence of biases in individuals who receive opiate substitution therapy (16). The presence of biases among individuals maintained on opiate substitution therapy implies that whilst pharmacological interventions might help in the stabilization of the lifestyle and minimisation of harms associated with illicit usage, it does not have any effect on factors that could still predispose individuals to a lapse or relapse (14). Drug-related stimuli have been proposed to be “cognitive intermediates” prior to a lapse or relapse, given that such salient stimuli activate unconscious processes, leading to one having increased attention, but also leaves the individual with fewer resources available to apply learned coping strategies. Hence, there is clearly a need for cognitive bias modification to be considered for individuals with addictive disorders. There has been increased recognition of this need, as evident by the protocol proposed by Heitman et al. (54), in which they attempt to investigate both the effectiveness and cost-effectiveness of an online Internet-based cognitive bias modification method that is delivered in addition to treatment as usual for individuals with alcohol or cannabis use disorders. Whilst there has been a prior study (55) that has evaluated combined cognitive bias modification and cognitive behavioral therapy, there remains, to our knowledge, no published studies or protocol for a similar study for substance use disorders. A consideration of the integration of both modalities of therapy is crucial, given that cognitive behavioral therapies typically target the top-down or reflective conscious decision-making processes, whilst bias modification could target the bottom-up or unconscious processes that are responsible for lapse and relapse occurrences.

The fact that cognitive biases could be detected by different tools negates the previous concerns raised about the reliability of the assessment tools. Biases were reported to be present despite there being a varied range of assessment tools that were utilized. The combination of indirect and direct measures used in the ascertainment of cognitive biases helped improve the evidence base, supporting the presence of these biases in all three disorders. Across all three disorders, a combination of both indirect and direct measures was used in the ascertainment of cognitive biases. Indirect measures, as aforementioned, refer to assessment tools such as the Stroop task or the visual probe task. In these indirect measures, participants are required to either name the color in which neutral and drug-related words are printed or respond to probes that replace either neutral or salient stimuli (53). Reaction time is measured and used as a surrogate in the determination of attentional biases. Direct measures provide better evidence of cognitive biases, given that eye movements in response to neutral or drug cures are used in the ascertainment of cognitive biases (12). Ataya et al. (56) previously investigated the internal reliability of the Stroop task and the visual probe task in the assessment of attention biases and reported that the modified Stroop task was more reliable as compared to the visual probe task, as it is a simpler task and is less likely to influence the reaction time that is being measured. Field and Christiansen (57) reported that the eye-movement measurement of cognitive bias does help to further mitigate against the concerns about reliability. Given that a variety of conventional indirect measures and direct measures were used for all the different disorders, we believe that this helps to better the quality of our synthesized evidence of the presence of cognitive biases in these disorders.

Notably, there was a single study Montgomery et al. (39) that has utilized different methods of cognitive bias assessment in the same group of participants. Interestingly, the authors reported that there was an increase in attentional biases following the administration of the visual probe task. However, participants' response did not differ when the modified Stroop task was used. Montgomery et al. (39) explained the differences in their results by highlighting that the visual-probe task might be better in measuring visuospatial attention. The Stroop task might not be as sensitive in the measurement of visuospatial attention but is better in assessing for response inhibition and other cognitive processes. Whilst we have previously discussed the advantages of having both direct and indirect measures in the assessment of biases, the results arising from Montgomery et al. 's (39) study has other implications if only indirect measures are to be used. This might be in the context of web or mobile-delivery of cognitive bias modification intervention. As such, it is of importance for researchers to carefully consider which assessment tool might be most appropriate, depending on the stimulus that they present to participants, given that the visual probe task is better able to measure visuospatial attention, and the Stroop task better for response inhibition. Also, while the Stroop task has been found to be more reliable, the results arising from Montgomery et al.'s (39) study also highlights the need for researchers to consider other aspects of the intervention, that might affect whether the task is capable of detecting biases.

This review has several strengths. A comprehensive search through the literature identified studies for the highly prevalent addictive disorders. No other reviews have synthesized the existing evidence for biases and cannabis use disorders. There are however limitations. The inclusion of a mixture of study designs, including both randomized and non-randomized studies affects the quality of the evidence, given that non-randomized studies have the risk of confounding and other biases. We were unable to perform a risk of bias assessment, due to the diversity of study designs and there remains no single tool that could assess risk of bias across different study designs. Whilst we search for studies involving participants with stimulant use disorders, the studies included involved participants who were primarily using cocaine. It will be beneficial if studies involving participants who were using amphetamines be conducted and incorporate into future synthesis, particularly as there is an increasing global trend of amphetamine abuse and dependence. We are also limited to a qualitative synthesis of the data extracted from each of the studies pertaining to attentional biases. Most of the studies included focus on the assessment of attentional biases in substance-using individuals, so evidence synthesis for the effectiveness of attentional or approach bias modification could not be undertaken. Secondary outcome measures had great heterogeneity, so we were unable to identify a common dataset for the synthesis.

## Conclusions

Cognitive biases have been consistently observed in opioid use, cannabis use, and stimulant use disorders, despite a range of assessment tools being utilized in the assessment for these biases. The presence of attentional and approach bias signifies the importance of bias modification interventions. There remains a need for future research to explore the presence of attentional biases in other stimulant use disorders, such as amphetamine use disorders. It is also of importance for future research to evaluate the efficacy of bias modification for these highly prevalent disorders and to determine if reduction of these biases is associated with improvements in other addiction outcomes.

## Data availability

All available data have been included in the manuscript.

## Author contributions

MZ, DF, and HS conceptualized and planned the current review. MZ and JY were involved in the literature search, extraction, and selection of the articles for inclusion in the current review. MZ wrote the initial draft of the manuscript and TW helped in the amendments of the initial draft. All authors were involved in the subsequent revisions and preparations of the manuscript prior to submission.

### Conflict of interest statement

The authors declare that the research was conducted in the absence of any commercial or financial relationships that could be construed as a potential conflict of interest.
